# Shank Proteins Differentially Regulate Synaptic Transmission

**DOI:** 10.1523/ENEURO.0163-15.2017

**Published:** 2017-12-15

**Authors:** Rebecca Shi, Patrick Redman, Dipanwita Ghose, Hongik Hwang, Yan Liu, Xiaobai Ren, Lei J. Ding, Mingna Liu, Kendrick J. Jones, Weifeng Xu

**Affiliations:** 1 Picower Institute for Learning and Memory; 2 Department of Brain and Cognitive Sciences; 3 Department of Biology; 4 Department of Chemistry, Massachusetts Institute of Technology, Cambridge, MA 02139

**Keywords:** ASD, bicuculline, excitatory, hippocampus, mini, Prosap1

## Abstract

Shank proteins, one of the principal scaffolds in the postsynaptic density (PSD) of the glutamatergic synapses, have been associated with autism spectrum disorders and neuropsychiatric diseases. However, it is not known whether different Shank family proteins have distinct functions in regulating synaptic transmission, and how they differ from other scaffold proteins in this aspect. Here, we investigate the role of Shanks in regulating glutamatergic synaptic transmission at rat hippocampal SC-CA1 synapses, using lentivirus-mediated knockdown and molecular replacement combined with dual whole-cell patch clamp in hippocampal slice culture. In line with previous findings regarding PSD-MAGUK scaffold manipulation, we found that loss of scaffold proteins via knockdown of Shank1 or Shank2, but not Shank3, led to a reduction of the number but not the unitary response of AMPAR-containing synapses. Only when both Shank1 and Shank2 were knocked down, were both the number and the unitary response of active synapses reduced. This reduction was accompanied by a decrease in NMDAR-mediated synaptic response, indicating more profound deficits in synaptic transmission. Molecular replacement with Shank2 and Shank3c rescued the synaptic transmission to the basal level, and the intact sterile α-motif (SAM) of Shank proteins is required for maintaining glutamatergic synaptic transmission. We also found that altered neural activity did not influence the effect of Shank1 or Shank2 knockdown on AMPAR synaptic transmission, in direct contrast to the activity dependence of the effect of PSD-95 knockdown, revealing differential interaction between activity-dependent signaling and scaffold protein families in regulating synaptic AMPAR function.

## Significance Statement

Postsynaptic scaffold proteins at the glutamatergic synapses include several specific families, of which, many genes are associated with neurodevelopmental and neuropsychiatric disorders. The functional significance and diversity of these scaffolds remain to be elucidated. Here, we investigate how scaffold proteins, Shanks, regulate hippocampal SC-CA1 synaptic transmission. We found loss of different Shank proteins led to different degrees of deficit in AMPAR-mediated synaptic transmission, with the unitary response of AMPAR-containing synapses prioritized to be maintained. Additionally, altered neural activity did not influence the effect of Shank knockdown on AMPAR synaptic transmission, in contrast to the effect of PSD-95 knockdown, indicating differential interaction between neuronal activity and scaffold proteins in regulating synaptic AMPAR function.

## Introduction

The postsynaptic density (PSD) comprises scaffold proteins that interact with each other to maintain the structural stability of the postsynaptic configuration, while organizing the receptor complexes and postsynaptic signaling cascades important for activity-dependent modification of mammalian glutamatergic synapses (Scannevin and Huganir, 2000; Kim and Sheng, 2004; Kennedy et al., 2005). Shank (SH3 and multiple ankyrin repeat domains protein) proteins are multidomain structural proteins enriched in the PSD of excitatory synapses (Naisbitt et al., 1999; Rostaing et al., 2006), forming a macro-molecular complex with other PSD-enriched molecules (Tu et al., 1999; Sheng and Kim, 2000; Grabrucker et al., 2011a). It has been hypothesized that, through this multitude of molecular interactions, Shank family proteins scaffold ionotropic and metabotrophic glutamate receptors to cytoskeletal components, thereby regulating synaptic morphology and synaptic function (Scannevin and Huganir, 2000; Kim and Sheng, 2004; Kennedy et al., 2005; Frost et al., 2010; Grabrucker et al., 2011b). Supporting this hypothesis, manipulating Shank family proteins results in changes in synapse development, spine structure, PSD organization, synaptic glutamate receptor levels and synaptic transmission (Sala et al., 2001; Grabrucker et al., 2011a). Three individual genes encode Shank family proteins: Shank1, Shank2, and Shank3. Shank1 has been proposed to be a master regulator of the synaptic scaffold (Ehlers, 2003). All Shanks have been associated with neurologic diseases such as schizophrenia and autism (Durand et al., 2007; Gauthier et al., 2010; Grabrucker et al., 2011a; Leblond et al., 2014), but the severity of the phenotype seems to be gene specific. It is not known whether different Shank family members have distinct or overlapping functions, and how they differ from other scaffold proteins in regulating synaptic transmission. Understanding their individual and distinct roles in regulating synaptic transmission could provide critical insight into mechanisms of glutamatergic synaptic function under normal and pathologic conditions.

Several lines of mice have been generated to genetically ablate specific Shank genes and/or their splice isoforms. These lines of mice show an array of phenotypes including defects in basal synaptic transmission (for review, see Jiang and Ehlers, 2013). However, the resulting phenotypes were not consistent with each other. This apparent inconsistency may be due to different targeting strategies, different brain regions and developmental stages analyzed, and possible developmental and activity-dependent compensation. To circumvent complications inherent to these approaches, we sought a different approach to compare the principal contributions of each Shank family protein in a systematic manner. We used a lentivirus-mediated gene knockdown to down-regulate the expression in hippocampal CA1 neurons in organotypic slice cultures, and then tested synaptic transmission at hippocampal Schaffer Collateral-CA1 synapses. With its defined structure, the hippocampus allows manipulation of postsynaptic proteins without influencing the target proteins in the presynaptic neurons. The organotypic slice culture allows dual whole-cell patch clamping to measure evoked excitatory synaptic transmission in adjacent infected and uninfected neurons stimulated by the same set of axonal afferents. Furthermore, the organotypic slice culture permits the use of chronic pharmacological treatment to study the interactions between neuronal activity and our molecular manipulations.

## Materials and Methods

### Virus preparation and infection

All lentiviral constructs were modified from the original lentiviral transfer vector FUGW (Lois et al., 2002), and its variant FHUG+W with an additional RNAi expression cassette driven by an H1 promoter (Schlüter et al., 2006). Lentiviral constructs were modified to target mRNA sequences of Shank1 (GGGTTGAAGAAGTTCCTTGAA), Shank2 (GGGCACAGGATGAACATAGAA), Shank3 (shShank3, CCCTCTTTGTGGATGTGCAAA, shShank3 alternative GGCCAGGAATGTTGCATGAAT in the 3’-UTR), or a common sequence between Shank1 and Shank3 mRNA (GACAAGGGGCTGGACCCCAAT). Constructs also contained ubiquitin promoter-driven eGFP or tdTomato (tdT), which allowed identification of infected cells. Superinfection with both eGFP and tdT viruses allowed multiple combinations of Shank knockdowns to be performed. Shank2 cDNA with silent mutations in the shShank2 targeting site (ggCcaTCgCatgaaTatCgaG) was fused to the C-terminus of eGFP in respective lentiviral vectors to construct replacement vectors. Shank3c cDNA was cloned in the similar fashion with no silent mutation introduced, as shShank13 targets the Shank3 sequence that is not present Shank3c isoform. To produce the lentiviruses, the transfer vectors and the HIV-1 packaging vectors (pRSV/REV, pMDLg/pRRE, and the VSV-G envelope glycoprotein vector (Dull et al., 1998) were cotransfected into HEK293T fibroblasts (ATCC, RRID: CVCL_0063) using the FUGENE6 transfection reagent (Promega). Supernatants of culture media were collected 60 hours after transfection, and then centrifuged at 50,000 × *g* to concentrate the viral particles.

### Dissociated cortical neuron cultures

Dissociated cortical cultures were prepared from P1 Sprague Dawley rat pups of either sex. The cortical hemispheres were dissected out and digested with papain for 20 min at 37°C, according to the protocol followed by (Schlüter et al., 2006). To infect cortical cultures, 1.5 μl of concentrated viral aliquot were dispensed into 2 ml of culture media per well of a 12-well plate, at 7 days in vitro (DIV7) and collected after DIV17. Cells were washed with ice-cold PBS, and lysed with homogenization buffer (4 mM HEPES, pH 7.4, 0.32 M sucrose, 2 mM EGTA, and protease inhibitors). The homogenate was centrifuged at 800 × *g* for 10 min at 4°C, after which the supernatant was centrifuged again at 10,000 × *g* for 15 min at 4°C. This second pellet (P2) was used for Western blot analyses.

### HEK293T fibroblast cultures

HEK293T fibroblasts were cultured in DMEM supplemented with 10% FBS, and transfected with a Shank3 expressing plasmid and either GFP, shShank1, shShank2, shShank3 or shShank13 expressing vectors. The cell lysates were collected in standard protein sample buffers 48 hours post transfection and subjected for Western blot analyses.

### Hippocampal slice cultures

Hippocampi of P7 Sprague Dawley rat pups of either sex were isolated and slice cultures were prepared following a published protocol (Liu et al., 2014). When slices were treated pharmacologically, 20 μM bicuculline (Tocris) or 25 μM D-APV (Tocris) was included in the media 2 d after virus injection, and bicuculline or D-APV was present until the day of recording. To infect hippocampal slice cultures, concentrated viral solutions were injected into the CA1 pyramidal cell layer using a Nanojector (Drummond). To achieve superinfection, lentivirus particles were super-concentrated at 4-fold. Equal volume of two different lentiviruses were mixed and coinjected.

### Western blotting

The following primary antibodies were used: anti-Shank1 (1:200, Abcam, catalog #ab154224), anti-Shank2 (1:100, Cell Signaling, catalog #12218), anti-Shank3 (1:400, Santa Cruz, RRID: AB_2301759), anti-PanShank (1:1000, Neuromab, RRID: AB_10674115), and anti-actin (1:3000, Sigma, RRID: AB_476697). IRDye 800CW and 680LT Secondary antibodies (Licor) were used at 1:5000 dilution for detection on an Odyssey IR laser Scanner (Licor). All anti-Shank signals were normalized to the actin signal. Data from infected neurons were compared to data from uninfected neurons within the same batch. Statistical significance was estimated with Student’s *t* test between infected and uninfected neuron cultures.

### Electrophysiology

All experiments were performed at 29-30°C, after slices had been infected for 5-8 d. Recording conditions followed from published studies (Liu et al., 2014). For evoked EPSC (eEPSC) recordings, neurons were recorded under voltage-clamp configuration in ACSF containing: 119 mM NaCl, 26 mM NaHCO_3_, 10 mM glucose, 2.5 mM KCl, 1 mM NaH_2_PO_4_, 4 mM MgSO_4_, and 4 mM CaCl_2_, saturated with 95% O_2_/5% CO_2_ and supplemented with 1μM 2-Chloroadenosine, 50 μM picrotoxin. The patch pipette (4.5–7 MΩ) solution contained: 115 mM CsMeSO_3_, 20 mM CsCl, 10 mM HEPES, 4 mM MgCl_2_, 4 mM NaATP, 0.4 mM NaGTP, 10 mM sodium phosphocreatine, 5 mM QX-314, and 0.5 mM EGTA, pH 7.3. For mini EPSC recordings, ACSF was additionally supplemented with 1μM tetrodotoxin, 50 μM D-APV and 50 mM sucrose. The patch pipette solution contained: 130 mM CsMeSO_3_, 20 mM CsCl, 10 mM HEPES, 6 mM MgCl_2_, 2 mM NaATP, 0.3 mM NaGTP, 5 mM sodium phosphocreatine, 5 mM QX-314, and 5 mM EGTA, pH 7.3. For both eEPSCs and mEPSCs, data were collected using a MultiClamp 700B amplifier (Molecular Devices), digitized at 10 kHz with the A/D converter ITC-18 computer interface (Heka Instruments). Data were acquired and analyzed on-line using custom routines written with Igor Pro software (Wavemetrics). Input and series resistances were monitored throughout the recordings. mEPSCs were analyzed off line with Mini Analysis Program (Synaptosoft) using a threshold of 6 pA.

For both eEPSC and mEPSC statistical analyses between pair-recorded uninfected and infected neurons, significance was estimated with a two-tailed, paired Student’s *t* test. The neurons from each pair were exposed to the same dissection, culture, and injection procedures (mEPSC and eEPSC), and the same stimulated afferent input (eEPSC), therefore paired analyses were used for these analyses to control the experimental conditions. Significance was determined at *p* < 0.05. When plotting eEPSCs ratios across experimental conditions, averages of ratios of infected and uninfected cell pairs were logarithmically transformed and presented as back-transformed mean ± SEM. Statistical significance from the described paired *t* test above was shown on top of the bar.

## Results

### Lentivirus-mediated knockdown of specific Shank family proteins

**Figure 1.**
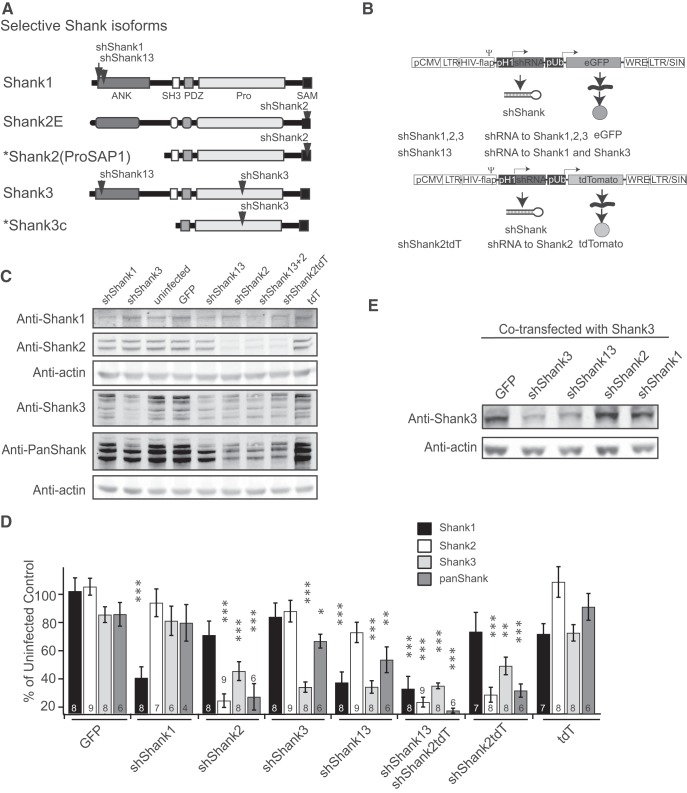
Acute knockdown of Shank family proteins via lentivirus-mediated shRNA expression. ***A***, Schematics of Shank proteins indicating shRNA target sites. ***B***, Schematics of lentiviral constructs used to introduce shRNAs into cells. Constructs contained one of four shRNAs targeting Shank1 (shShank1), Shank2 (shShank2), Shank3 (shShank3), or Shank1 and Shank3 simultaneously (shShank13), as well as one of two fluorescent proteins (GFP, green fluorescent protein; tdT, tdTomato). pCMV, cytomegalovirus promoter; LTR, long terminal repeats; HIV-flap, a nuclear import sequence; pH1, constitutive H1 promoter; pUb, constitutive ubiquitin promoter; WRE, woodchuck hepatitis virus post-transcriptional regulatory element. ***C***, ***D***, Example (***C***) and quantification (***D***) of Western blotting for Shank protein levels in dissociated cortical neuron culture. Actin was used as a loading control. GFP and tdT refer to cultures infected with virus constructs containing the fluorescent protein and no shRNA. shShank13 + 2, knockdown of all three Shank members by superinfection of shShank13 and shShank2. ***E***, A Western blotting for Shank3 levels in HEK cells cotransfected with a rat Shank3 expression vector and a GFP or shRNA expressing construct as indicated. One-way ANOVA was used for each quantification, followed by Tukey’s test, *p < 0.05; **p < 0.01; ***p < 0.001.

To determine the role of Shank proteins in maintaining synaptic transmission, we used a lentivirus-mediated shRNA knockdown to reduce Shank levels in neurons. For our knockdown experiments, we designed lentiviral constructs containing shRNAs targeting either rat Shank1 (shShank1), Shank2 (shShank2), Shank3 (shShank3), or an shRNA targeting both Shank1 and Shank3 (shShank13). We screened five to eight shRNA sequences for each of the Shank genes, and identified at least one effective shRNA construct for each of the Shank genes. The targeting regions of the effective shRNAs in selective Shank isoforms are shown in Figure 1*A*. The constructs also expressed a fluorescent protein such as eGFP or tdT to allow visual identification of infected cells (Fig. 1*B*).

To confirm the specificity and efficacy of the shRNAs, we infected dissociated cortical neuron cultures with lentiviruses containing the shRNAs. Infected cultures were analyzed using Western blotting to detect Shank1, Shank2, and Shank3 levels in the synaptoneurosome fraction (P2), using actin as a loading control (Fig. 1*C*). A panShank antibody was also used to assay overall Shank protein levels, with the caveat that the affinity of the PanShank antibody to different Shank isoforms was unknown. Quantification of the blots showed that infection with control viruses (labeled as GFP or tdT) containing the H1 promoter but not specific shRNAs did not reduce Shank protein levels in the synaptosomal fraction as compared to uninfected cultures, indicating viral infection alone had little effect on the expression of Shank proteins. As expected, infection with shShank1 or shShank3 viruses reduced only the levels of their respective target proteins. Similarly, infection with the shShank2 viruses effectively reduced the levels of Shank2 (Fig. 1*D*). Interestingly, shShank2 expression also led to small but significantly reduced levels of Shank3.

To test whether this decrease of Shank3 expression by shShank2 was due to an off-target effect, we cotransfected human embryonic kidney (HEK) cells with a full-length rat Shank3-expressing construct and various shRNA expressing constructs. Only coexpression with Shank3 targeting shRNAs (shShank3 and shShank13) reduced HEK cell expression of Shank3, but not coexpression of shShank1, shShank2 or GFP (Fig. 1*E*), suggesting shShank2 does not have an off-target effect on Shank3 expression. The decrease of Shank3 levels accompanying shShank2 expression is thus most likely a functional consequence of reduced Shank2 levels in the synapses.

We also designed an shRNA targeting a common sequence shared by Shank1 and Shank3. The virus expressing this shRNA (shShank13) effectively reduced the levels of Shank1 and Shank3 but not that of Shank2 (Fig. 1*B*). Finally, superinfecting the neurons with shShank13 and shShank2 (shShank13 + 2) significantly decreased levels of all three Shanks as expected (Fig. 1*C*,*D*). This superinfection allowed us to assay the functional consequence of decreasing most if not all Shank proteins in synaptic transmission.

### Knockdown of Shank1 or Shank2 reduces AMPAR-mediated synaptic transmission by reducing the number of active synapses

To determine the effect of Shank knockdown on synaptic transmission, we injected shRNA-containing lentiviruses into the CA1 region of cultured hippocampal slices and recorded excitatory postsynaptic currents (EPSCs) from CA1 pyramidal cells. We recorded simultaneously from one infected cell and a neighboring uninfected cell to directly compare their responses to the same stimulation. It has been shown that lentivirus-mediated expression of GFP or other fluorescent proteins does not influence basal EPSCs (Nakagawa et al., 2004; Elias et al., 2006; Schlüter et al., 2006). In an additional control experiment, we verified that AMPAR-mediated eEPSCs (AMPAR eEPSCs) and NMDAR-mediated eEPSCs (NMDAR eEPSCs) were not affected in cells superinfected with GFP and tdT viruses that contained the H1 promoter cassette without effective shRNAs (AMPAR eEPSCs, *n* = 12 pairs, control, −45.5 ± 5.7 pA; infected, −46.0 ± 15.7 pA, *p* = 0.97; NMDAR eEPSCs, *n* = 11 pairs, control, 21.0 ± 3.3 pA, infected, 20.6 ± 3.0 pA, *p* = 0.83, graph not shown).

**Figure 2.**
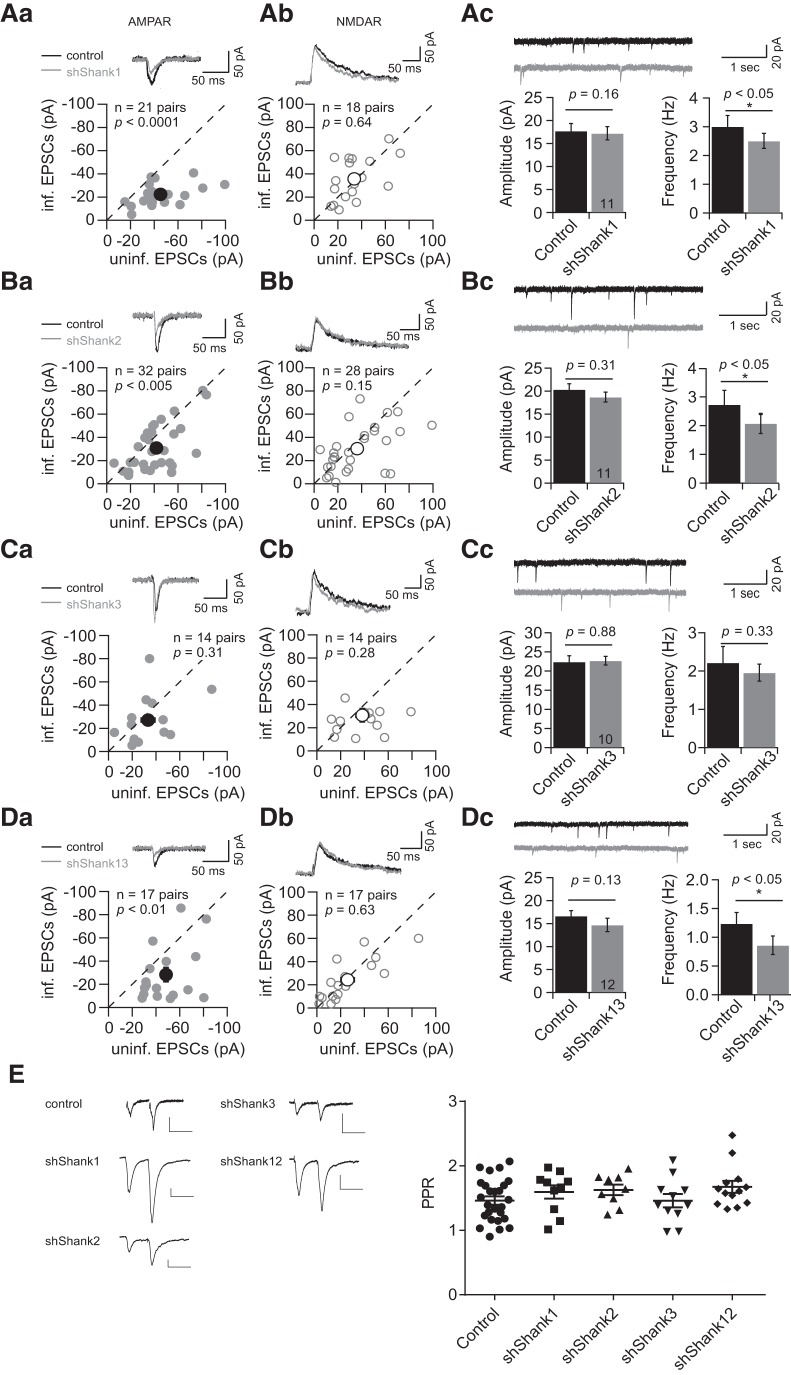
Knockdown of Shank1 or Shank2 decreased AMPAR-mediated currents by reducing active synapse number. ***A–D***, Comparison of uninfected (control) and infected (***A***, shShank1; ***B***, shShank2; ***C***, shShank3; ***D***, shShank13) neuronal responses measured by eEPSCs-( individual data point, ● mean, AMPAR eEPSCs (***Aa–Da***, left panels); individual data point, ○ mean, NMDAR eEPSCs (***Ab–Db***, middle panels)] and mEPSCs (***Ac–Dc***, right panels), example traces (top of each panel)]. Error bars ± SEM. Student’s paired *t* test was used for data analyses. Scale bars: 50 ms by 50 pA. ***Ac–Dc***, Amplitudes of mEPSCs (left panels) and frequencies of mEPSCs (right panels). Bar graphs, mean ± SEM. Student’s paired *t* test was used for data analyses; *p < 0.05. The data presentation, quantification, and statistical analyses were the same in the following figure unless indicated otherwise. ***E***, left panels, Example traces of PPP (50-ms interval) measured from indicated neurons. Scale bar: 20 pA, 50 ms. Right, Summary of PPR from uninfected neurons (●, *n* = 26, 1.46 ± 0.07); shShank1-infected neurons (■, *n* = 10, 1.59 ± 0.10); shShank2-infected neurons (▲, *n* = 9, 1.63 ± 0.08); shShank3-infected neurons (▼, *n* = 11, 1.46 ± 0.10); shShank1 + shShank2-infected neurons (♦, *n* = 13, 1.67 ± 0.09).

The expression of shShank1 reduced AMPAR eEPSCs but not NMDAR eEPSCs (shShank1, AMPAR eEPSCs, *n* = 21 pairs, control, −45.2 ± 4.5 pA; infected, −22.4 ± 2.0 pA, *p* < 0.0001; NMDAR eEPSCs, *n* = 18 pairs, control, 33.7 ± 4.1 pA, infected, 35.7 ± 4.3 pA, *p* = 0.64; Fig. 2*Aa*,*Ab*). To determine whether the decrease in AMPAR eEPSCs is due to a decrease in the number of AMPAR-containing synapses (active synapses), or a decrease in the unitary strength of active synapses, we measured AMPAR-mediated excitatory miniature EPSCs (AMPAR mEPSCs). Our results show that knockdown of Shank1 reduced mEPSC frequency but not mEPSC amplitude (shShank1, *n* = 11 pairs; amplitude, control, 17.8 ± 1.6 pA; infected, 17.2 ± 1.5 pA, *p* = 0.16; frequency, control, 3.0 ± 0.4 Hz, infected, 2.5 ± 0.3 Hz, *p* < 0.05; Fig. 2*Ac*). The mEPSC frequency can be influenced by both the number of AMPAR-containing active synapses and the presynaptic release probability. The paired-pulse ratio (PPR), which can be used to estimate the presynaptic release probability, was not significantly different between uninfected and infected neurons (Fig. 2*E*). Thus, our data indicate that decreasing Shank1 levels decreases AMPAR-mediated synaptic transmission through a reduction of the number of active synapses, without significantly affecting unitary synaptic strength.

Similar to shShank1, decreasing Shank2 expression levels with shShank2 also reduced AMPAR eEPSCs but not NMDAR eEPSCs (AMPAR eEPSCs, *n* = 32 pairs, control, −41.7 ± 3.3 pA, infected, −30.8 ± 3.5 pA, *p* < 0.005; NMDAR eEPSCs, *n* = 28 pairs, control, 36.3 ± 4.5 pA, infected, 30.3 ± 3.7 pA, *p* = 0.15; Fig. 2*Ba*,*Bb*), reduced mEPSC frequency but not mEPSC amplitude (*n* = 11 pairs, amplitude, control, 20.4 ± 1.2 pA, infected, 18.7 ± 1.1 pA, *p* = 0.31; frequency, control, 2.7 ± 0.5 Hz, infected, 2.1 ± 0.4 Hz, *p* < 0.05; Fig. 2*Bc*), with no difference in PPR between uninfected and infected neurons (Fig. 2*E*). Thus, decreasing either Shank1 or Shank2 levels decreases AMPAR-mediated synaptic transmission through a reduction of the number of active synapses.

In contrast to Shank1 or Shank2 knockdown, Shank3 knockdown had no effect on either AMPAR or NMDAR eEPSCs (shShank3, *n* = 14 pairs; AMPAR eEPSCs, control, −33.3 ± 5.6 pA; infected, −27.1 ± 4.1 pA, *p* = 0.31; NMDAR eEPSCs, control, 38.2 ± 5.2 pA, infected, 30.6 ± 5.6 pA, *p* = 0.28; Fig. 2*Ca*,*Cb*) and also did not affect mEPSCs (shShank3, *n* = 10 pairs; amplitude, control, 22.4 ± 1.6 pA; infected, 22.7 ± 1.1 pA, *p* = 0.33; frequency, control, 2.2 ± 0.4 Hz, infected, 2.0 ± 0.2 Hz, *p* = 0.88; Fig. 2*Cc*). An alternative shRNA to Shank3 with similar knockdown efficiency was also used and produced similar results (shShank3_2, *n* = 12 pairs; AMPAR eEPSCs, control, −38.1 ± 6.8 pA; infected, −32.4 ± 5.4 pA, *p* = 0.38; NMDAR eEPSCs, control, 37.7 ± 11.01 pA, infected, 44.2 ± 12.5 pA, *p* = 0.69). Furthermore, simultaneous knockdown of Shank1 and Shank3 (shShank13) produced results similar to those of shShank1 (shShank13, *n* = 17 pairs; AMPAR eEPSCs, control, −48.5 ± 4.5 pA; infected, −28.4 ± 5.9 pA, *p* < 0.01; NMDAR eEPSCs, control, 25.8 ± 5.5 pA, infected, 24.2 ± 4.4 pA, *p* = 0.63; mini AMPAR EPSCs, *n* = 10 pairs; amplitude, control, 16.7 ± 1.2 pA; infected, 14.7 ± 1.5 pA, *p* = 0.13; frequency, control, 1.2 ± 0.2 Hz, infected, 0.9 ± 0.2 Hz, *p* < 0.05; Fig. 2*D*). These results suggest that reducing Shank3 levels has little effect on synaptic transmission at Schaffer collateral-CA1 synapses in our experimental conditions.

### Simultaneous knockdown of Shank1 and Shank2 further decreases synaptic transmission with decreased unitary active synapse response and NMDAR-mediated response

**Figure 3.**
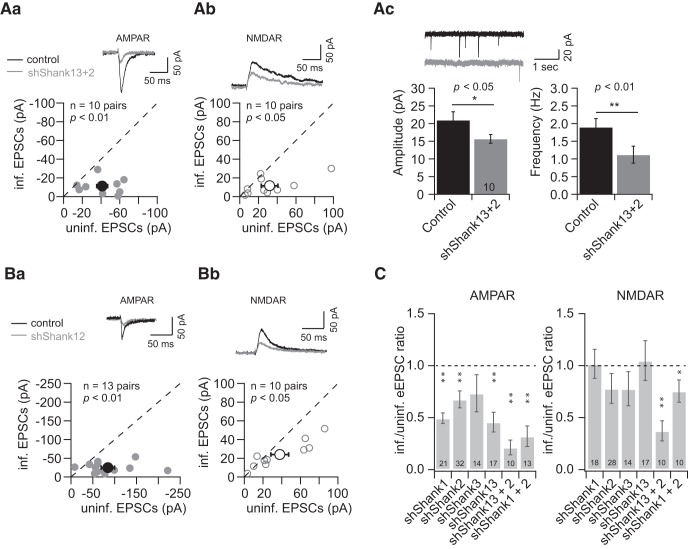
Simultaneous knockdown of Shank1 and Shank2 decreased both AMPAR- and NMDAR-mediated currents. ***A***, ***B***, Comparison of uninfected (control) and infected (***A***, shShank13 + 2; ***B***, shShank1 + 2) neuronal responses measured by eEPSCs [AMPAR eEPSCs (***Aa***, ***Ba***, left panels); NMDAR eEPSCs (***Ab***, ***Bb***, middle panels), and mEPSCs (***Ac***, right panel)]. ***C***, Summary of effects on AMPAR EPSCs (left panel) and NMDAR EPSCs (right panel) of knockdown of Shank1, Shank2, Shank3, Shank13 + 2, Shank1 + 2, mean ± SEM.

To further investigate the role of Shanks in synaptic transmission, we knocked down all three Shanks using a superinfection of shShank13 and shShank2. shShank13 was expressed in a construct with eGFP, while shShank2 was expressed with the red fluorescent protein, tdT. When all three Shanks were knocked down, we saw a decrease in both AMPAR and NMDAR eEPSCs (shShank13 + 2, *n* = 10 pairs; AMPAR eEPSCs, control, −41.2 ± 5.9 pA; infected, −11.2 ± 2.5 pA, *p* < 0.01; NMDAR eEPSCs, control, 31.8 ± 8.9 pA, infected, 11.7 ± 3.0 pA, *p* < 0.01; Fig. 3*Aa*,*Ab*). Furthermore, both mEPSC frequency and amplitude were decreased (shShank13 + 2, *n* = 10 pairs; amplitude, control, 21.0 ± 2.4 pA; infected, 15.7 ± 1.2 pA, *p* < 0.05; frequency, control, 1.9 ± 0.2 Hz, infected, 1.1 ± 0.2 Hz, *p* < 0.01; Fig. 3*Ac*). These results show that knockdown of all three Shanks leads to a loss of both the number and the unitary strength of active synapses, and that this severe loss of Shank scaffolding is sufficient to lead to a loss of NMDAR-mediated response.

Since Shank3 knockdown did not affect synaptic transmission when Shank1 and Shank2 were intact, we asked whether the effect of shShank13 + 2 on synaptic transmission was primarily due to the loss of Shank1 and Shank2. We achieved a double knockdown by expressing shShank1 in a construct with eGFP while expressing shShank2 with tdT, which allow us to identify double knockdown cells as both green and red. When both Shank1 and Shank2 were knocked down, both AMPAR and NMDAR eEPSCs were still decreased (shShank1 + 2, AMPAR eEPSCs, *n* = 13 pairs, control, −84.6 ± 15.3 pA; infected, −24.9 ± 3.6 pA, *p* < 0.01; NMDAR eEPSCs, *n* = 10 pairs, control, 38.0 ± 9.4 pA, infected, 24.0 ± 4.6 pA, *p* < 0.05; Fig. 3*B*), similar to shShank13 + 2. PPR was not affected by the superinfection (Fig. 2*E*). Collectively, these results indicate that Shank1 and Shank2 are the two principal Shank family scaffold proteins maintaining the synaptic transmission in hippocampal CA1 neurons in our experimental conditions (Fig. 3*C*).

### Altered neuronal activity does not influence the reduction of AMPAR eEPSCs caused by Shank1 and Shank2 knockdown

It has been shown previously, in hippocampal slice culture, that knockdown of the prominent scaffold protein PSD-95 produced similar effects to knockdown of Shank1 or Shank2. In particular, AMPAR eEPSCs were decreased but NMDAR eEPSCs were unaffected (Schlüter et al., 2006), and mEPSC frequency but not amplitude was decreased (Liu et al., 2014). Treating the hippocampal slice culture with bicuculline could rescue the decrease of AMPAR eEPSCs caused by knockdown of PSD-95 (Schlüter et al., 2006; Liu et al., 2014). Because bicuculline increases excitatory drive among neurons by blocking inhibitory synaptic transmission, these results indicate that the decrease of AMPAR mediated synaptic transmission caused by PSD-95 knockdown can be rescued in an activity-dependent manner.

**Figure 4.**
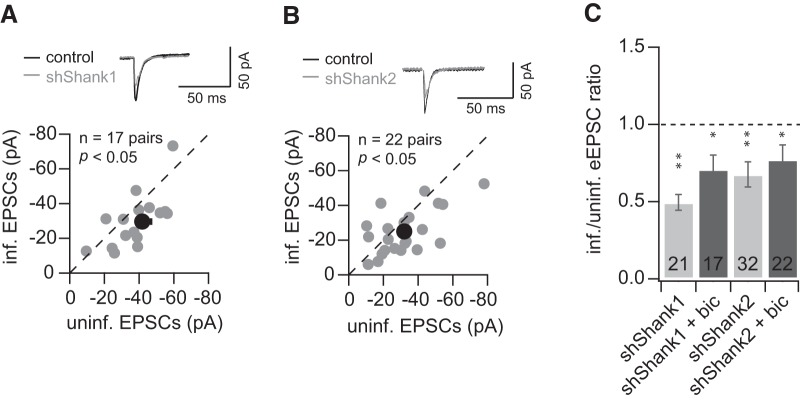
Enhancing excitatory drive does not rescue the decrease in AMPAR eEPSC caused by Shank knockdown. ***A***, ***B***, Comparison of uninfected (control) and infected [shShank1 (***A***); shShank2 (***B***)] neuronal responses measured by evoked AMPAR eEPSCs (● individual data point, ● mean) with bicuculline (20 μM). ***C***, Summary of effects on AMPAR eEPSCs of knockdown of Shank1 or Shank2 under control (light gray, data from Fig. 2, the same as in ***C*** as a comparison), with bicuculline (dark gray), mean ± SEM.

To test whether the decrease in AMPAR-mediated transmission caused by Shank knockdown is similarly regulated by neuronal activity, we treated hippocampal slices with pharmacological reagents for several days before electrophysiological recordings. In slices treated with bicuculline and infected with viruses containing either shShank1 or shShank2, AMPAR eEPSC amplitudes in infected neurons were still decreased compared to those in the neighboring uninfected neurons (shShank1, *n* = 17 pairs, control, −41.8 ± 5.1 pA; infected, −29.6 ± 3.7 pA, *p* < 0.05; shShank2, *n* = 22 pairs, control, −32.0 ± 3.6 pA; infected, −25.0 ± 2.8 pA, *p* < 0.05; Fig. 4*A*,*B*). Activity-dependent rescue of AMPAR eEPSCs following PSD-95 knockdown was still observed in parallel sister cultures (data not shown). These results indicate that elevating excitatory neuronal activity in slice culture does not influence the reduction of AMPAR eEPSCs caused by acute knockdown of Shank1 and Shank2 (Fig. 4*C*), unlike the activity-dependent effects of PSD-95 knockdown on AMPAR eEPSCs. These results indicate a functional divergence between PSD-95 and Shank family proteins in response to activity-dependent signaling pathways.

### The C-terminal SAM domain is critical for the effect of Shank2 on synaptic transmission

To further investigate the molecular mechanism underlying Shank-dependent regulation of synaptic transmission, we used a lentiviral molecular replacement vector (Schlüter et al., 2006) to overexpress Shank2 with simultaneous expression of shShank2, and examined the effects of Shank2 replacement on Shank levels and synaptic transmission. To allow for expression of recombinant Shank2 in the same cells expressing shShank2, the shShank2 target sequence in the recombinant Shank2 was silently mutated. The prototypic isoform of Shank2 is smaller compared to other isoforms of Shanks (Fig. 1*A*,*C*), which allowing to fit the coding region fused to GFP in the lentiviral molecular replacement vector.

**Figure 5.**
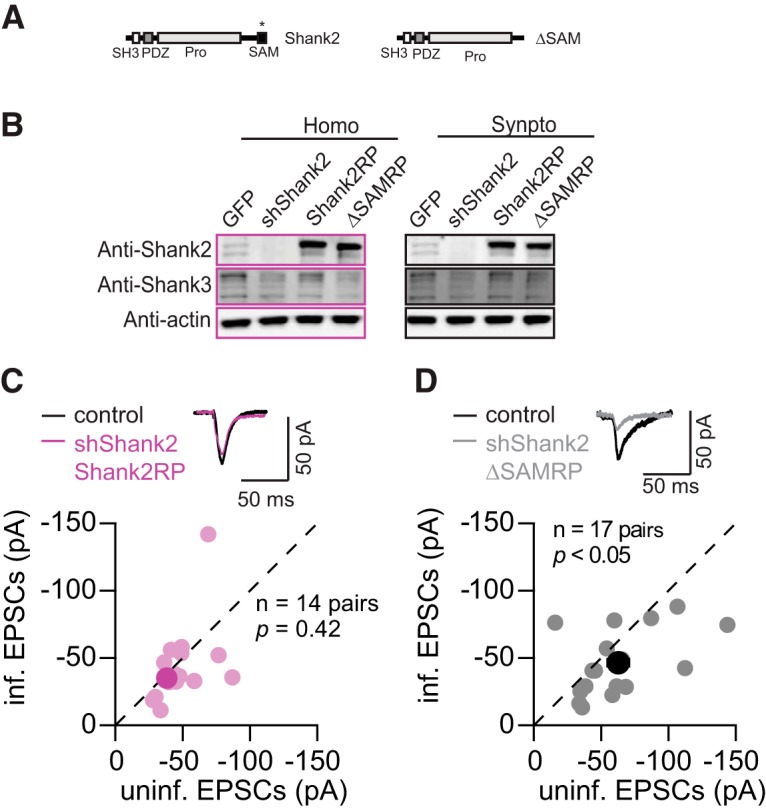
The SAM domain was required for Shank2 to maintain AMPAR eEPSCs. ***A***, Schematics of the domain structure of Shank2 and Shank2ΔSAM in the replacement construct. Silent mutations in Shank2 were indicated with *. ***B***, Examples of Western blotting for Shank2 and Shank3 protein levels in the total cell homogenate and the synaptoneurosome fraction from dissociated neuronal cultures infected with GFP, Shank2 replacement (Shank2RP), and Shank2ΔSAM replacement (ΔSAMRP). ***C***, ***D***, Comparison of uninfected (control) and infected [Shank2RP (**C**); ΔSAMRP (***D***)] neuronal responses measured by AMPAR eEPSCs (Shank2RP, individual data point, mean; ΔSAMRP, individual data point, ● mean).

We further used the molecular replacement system to examine the functional role of certain Shank protein domains. In particular, previous studies have shown that the C-terminal sterile α-motif (SAM) domain is critical for multimerization of Shank proteins (Naisbitt et al., 1999), and for synaptic localization of Shank2 and Shank3 (Boeckers et al., 2005). We therefore generated a Shank2 mutant lacking the SAM domain (ΔSAM; Fig. 5*A*) to test whether the SAM domain is important for the synaptic effects of Shank2.

In infected cortical cultures, both molecular replacement viruses efficiently silenced endogenous Shank2 while expressing recombinant GFP-tagged proteins at much higher levels comparable to the endogenous Shank2 levels in GFP-only expressing cultures (Fig. 5*B*). We also examined the levels of Shank3 in the synaptoneurosomal fraction from cultures infected with the replacement viruses. Replacing endogenous Shank2 with the wild-type Shank2 rescued the decrease of Shank3 levels seen with shShank2 expression (Figs. 1*C*,*D*, 5*B* ). This observation (Fig. 5*B*) indicates that the decrease of Shank3 with shShank2 expression was due to insufficient Shank2 levels.

Next, we tested the effect of Shank2 and ΔSAM replacement of endogenous Shank2 on synaptic transmission. Replacement with a wild-type Shank2 rescued AMPAR eEPSCs to the control level (shShank2 to Shank2 replacement, *n* = 14 pairs, control, −37.8 ± 3.2 pA; infected, −34.8 ± 3.8 pA, *p* = 0.42; Fig. 5*C*). AMPAR mEPSC frequency was rescued with no changes in mEPSC amplitude (shShank2 to Shank2 replacement, *n* = 8 pairs; amplitude, control, 18.8 ± 1.6 pA; infected, 14.7 ± 1.1 pA, *p* = 0.11; frequency, control, 1.8± 0.3 Hz, infected, 1.5 ± 0.3 Hz, *p* = 0.20). Despite our observation that exogenous Shank2 was expressed at a much higher level than the endogenous Shank2 level, AMPAR eEPSCs were rescued only to the control levels, not higher. This result indicates that this isoform of Shank2 is sufficient for maintaining basal synaptic AMPAR levels, but other factors are required for further enhancing the strength of AMPAR-mediated synaptic responses.

In contrast, replacement with ΔSAM did not rescue the decrease in AMPAR eEPSCs caused by shShank2 (Shank2 to ΔSAM replacement, *n* = 17 pairs; AMPAR eEPSCs, control, −62.7 ± 8.0 pA; infected, −46.5 ± 6.0 pA, *p* = 0.04; Fig. 5*D*). This result indicates that the SAM domain is important for mediating the effect of Shank2 on synaptic response, presumably via multimerization of Shank proteins (Naisbitt et al., 1999; Boeckers et al., 2005). Maintaining synaptic Shank2 levels with intact SAM domains in CA1 neurons is thus important for proper glutamatergic synaptic transmission and maintaining Shank levels in the synaptic compartment at the analyzed developmental stage.

### Short isoform of Shank3 rescues synaptic deficit caused by knocking down Shank1 or Shank2

**Figure 6.**
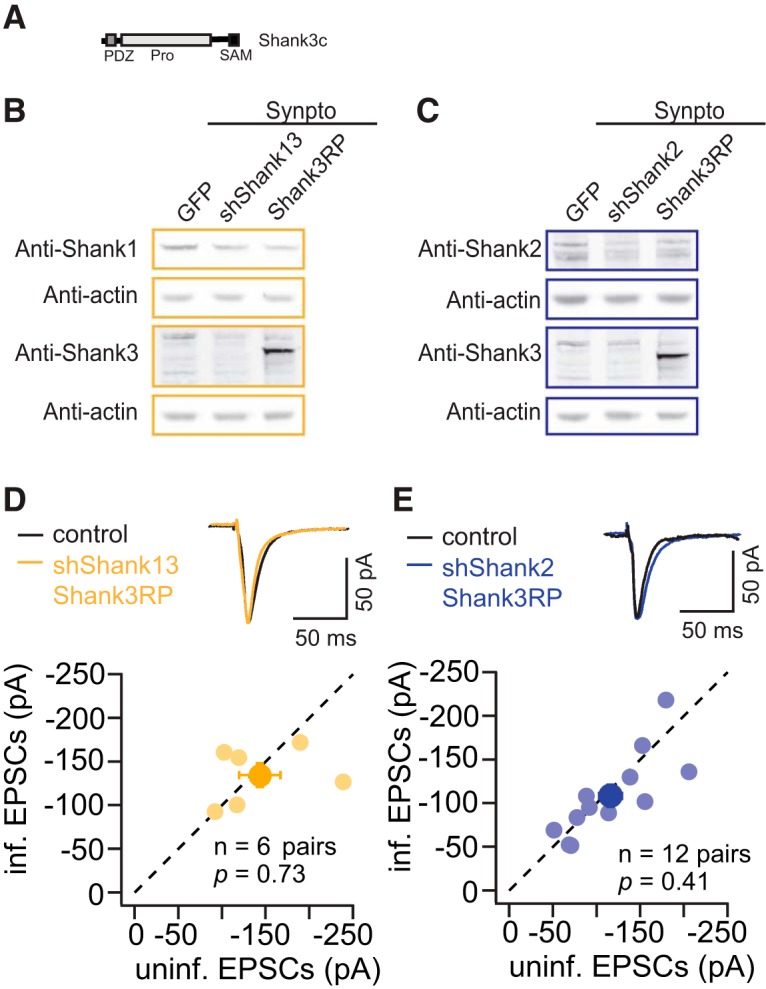
Shank3c rescues synaptic deficit caused by knocking down Shank1 or Shank2. ***A***, Schematics of the domain structure of Shank3c in the replacement construct. ***B***, Examples of Western blotting for Shank2 and Shank3 protein levels in the total cell homogenate and the synaptoneurosome fraction from dissociated neuronal cultures infected with GFP, shShank13, Shank3c replacement (Shank3RP). ***C***, Examples of Western blotting for Shank2 and Shank3 protein levels in the total cell homogenate and the synaptoneurosome fraction from dissociated neuronal cultures infected with GFP, shShank2, Shank3c replacement (Shank3RP). ***D***, Comparison of uninfected (control) and infected (shShank13 Shank3RP) neuronal responses measured by AMPAR eEPSCs ( individual data point, mean). ***E***, Comparison of uninfected (control) and infected (shShank2 Shank3RP) neuronal responses measured by AMPAR eEPSCs ( individual data point, mean).

Although shShank3 had no significant effect on the excitatory synaptic transmission in hippocampal slice cultures, it is likely that Shank3 can sufficiently support excitatory synaptic transmission. We overexpressed a Shank3 isoform Shank3c (Fig. 6*A*), similar to the prototypic Shank2 isoform, in the background of shShank2, and shShank13, and examined the effects of Shank3 replacement on Shank levels and synaptic transmission. As expected, in infected cortical cultures, both molecular replacement viruses efficiently silenced perspective endogenous Shank targets. GFP-tagged Shank3c proteins were expressed at much higher levels comparable to the endogenous Shank3 levels in GFP-only expressing cultures (Fig. 6*B*,*C*).

Next, we tested the effect of Shank3c replacement of endogenous Shank1 and Shank3 (shShank13) or Shank2 (shShank2) on synaptic transmission. Replacement Shank1 and Shank3 with Shank3c rescued AMPAR eEPSCs to the control level (shShank13 to Shank3 replacement, *n* = 6 pairs, control, −143.1 ± 23.7 pA; infected, −134.4 ± 13.5 pA, *p* = 0.73; Fig. 6*D*). In addition, replacement Shank2 with Shank3c rescued AMPAR eEPSCs to the control level (shShank2 to Shank3 replacement, *n* = 12 pairs, control, −116.1 ± 13.1 pA; infected, −108.4 ± 12.8 pA, *p* = 0.41; Fig. 6*E*). Similar the Shank2 replacement experiments, despite that exogenous Shank3 was expressed at a high level, AMPAR eEPSCs were rescued only to the control levels, not higher. These results indicate that Shank protein levels are required for maintaining basal synaptic AMPAR levels. The short isoforms of Shanks are sufficient for maintaining the basal synaptic function, but are not the rate limiting factor for further enhancing the strength of AMPAR-mediated synaptic responses.

## Discussion

In this study, we explored the role of the Shank family PSD scaffold proteins in regulating synaptic transmission at hippocampal Schaffer Collateral-CA1 synapses in the organotypic slice culture preparation. Our work lends functional support to the role of Shanks as critical proteins in the PSD scaffold (Naisbitt et al., 1999; Ehlers, 2003; Romorini et al., 2004; Rostaing et al., 2006). It has been shown that PSD-95 and Shank proteins are assembled together via SAPAP family proteins (Romorini et al., 2004), and that this tri-partner interaction is the core component of the PSD (Chen et al., 2008). Our studies show that the effect of knocking down Shank1 or Shank2 on synaptic transmission is similar to the effect of knocking down the PSD-MAGUK family proteins, with significant impact on the number of AMPAR-containing synapses, rather than the quantal size (Béïque et al., 2006; Elias et al., 2006; Ehrlich et al., 2007; Liu et al., 2014; Levy et al., 2015). These parallel observations point toward a general mechanism: when scaffold components are limited, neurons prioritize to maintain unitary synaptic strength of remaining active synapses at the expense of the number of active synapses (Levy et al., 2015). This preferential maintenance of synaptic strength in a subpopulation of active synapses suggests that a selection process may be at play. It remains unknown whether the signaling cascade including L-type calcium channels, CaM kinase activity and the GriA2 AMPAR subunit, involved in the synapse consolidation seen with PSD-MAGUK manipulation (Levy et al., 2015) is also at play with Shank manipulation.

Double knockdown of Shank1 and Shank2 and triple knockdown of all Shanks led to new phenotypes in synaptic transmission, including decreased unitary synaptic AMPAR mediated response measured by mini amplitudes, in addition to a profound decrease in numbers of active synapses, and also decreased NMDAR eEPSC responses, suggesting an essential role of Shank proteins for maintenance of glutamatergic synaptic transmission. Knocking down Shank3 had little effect on synaptic transmission in our experimental paradigm. This finding agrees with results from some Shank3 mutant lines tested in the hippocampus of juvenile animals (Peça et al., 2011; Wang et al., 2011), at a similar developmental stage to our preparation. It is possible that the lack of effect of Shank3 is due to different expression levels of Shank proteins at hippocampus and/or a potential dominant effect of Shank1 and Shank2 on regulating synaptic transmission at this developmental stage in hippocampal slice cultures. At striatal synapses (Peça et al., 2011) or adult hippocampal synapses (Yang et al., 2012), Shank3 may play a more important role in regulating synaptic AMPAR function.

Further studies need to be done to determine how the factors including gene-dosage, different knockdown methods (Levy et al., 2015), different developmental stage and different brain region may influence the effect of manipulation of Shank proteins on excitatory synaptic transmission. Different knockdown methods and the relative amount of endogenous proteins in different brain regions and at different developmental stage may influence the protein depletion rate and efficiency, which can potentially influence the effect on synaptic transmission.

Although we observed a synaptic phenotype at basal neural activity levels with manipulation of Shank family proteins similar to manipulations of PSD-MAGUK family proteins (Figs. 2, 4; Elias et al., 2006; Schlüter et al., 2006), Shank proteins are functionally distinct from other scaffold proteins in terms of activity-dependent regulation of synaptic transmission. In particular, the decreased AMPAR eEPSCs resulting from Shank knockdown could not be rescued with increased excitatory neuronal activity, unlike activity-dependent rescue of PSD-95 knockdown (Schlüter et al., 2006; Liu et al., 2014). Blocking NMDAR activity with D-APV also did not influence the effect of shShank2 on synaptic transmission (data not shown), indicating these alterations in neuronal activity do not play a significant role in Shank2-dependent regulation of AMPAR-mediated synaptic transmission. It is possible that Shank family proteins serve as the structural core of the scaffold, and the lack of Shank proteins cannot be compensated by activity-dependent AMPAR trafficking and interaction with PSD-MAGUKs. Alternatively, bicuculline- and D-APV-induced signaling events are specific for PSD-MAGUK family proteins, while Shank family proteins are targeted via other signaling cascades.

Shank2 and Shank3c were sufficient to rescue the synaptic deficit caused by decreasing Shank proteins, whereas Shank2ΔSAM, a mutant that was previously shown to perturb synaptic localization and functions of Shank2 (Naisbitt et al., 1999; Boeckers et al., 2005), was not sufficient. Together, these results suggest SAM domain-mediated interactions may play an important role in stabilizing synaptic scaffolds and exerting the effects of Shank2 on synaptic transmission.

In conclusion, we have shown the importance of Shank proteins in regulating synaptic transmission, demonstrating the functional divergence of Shank family members from each other and from PSD-MAGUK scaffold proteins in the hippocampal SC-CA1 synapse.

## Synthesis

Reviewing Editor: Christian Hansel, University of Chicago Decisions are customarily a result of the Reviewing Editor and the peer reviewers coming together and discussing their recommendations until a consensus is reached. When revisions are invited, a fact-based synthesis statement explaining their decision and outlining what is needed to prepare a revision will be listed below. The following reviewer(s) agreed to reveal their identity: Tobias M. Boeckers, Lu Chen.

The following issues are required to be addressed for a resubmission. I am also appending reviewer's comments at the end of this list, containing additional comments that authors might choose to address.

1. Authors should provide an explanation for the difference observed upon coexpressing shShank2 with GFP or td-Tomato.

2. Rescue experiments with small Shank3 isoforms (e.g., Shank3c, Shank3d) should be performed to aid in the interpretation of changes induced by sh-Shank2 expression. It would also be useful to determine if Shank2 can rescue the defects observed in Sh-Shank1.

3. Western blots or immunocytochemistry probing for potential changes in AMPAR levels should be included for in those conditions that alter AMPAR-mediated activity .

4. Besides measurements of spontaneous release frequency, measurements of probability of release should be conducted.

5. Authors should discuss the discrepancy between downregulation of Shank1 and Shank2 with regard to their effect on PSD95 and other non-PSD95 related potential mechanisms.

Minor issues:

1. Please carefully inspect the citation list, making sure that there is correspondence with citations within the text.

2. The diagram in Figure 1A is not accurate as it neglects the depiction of alternative isoforms

3. All Shank genes have been involved in psychiatric disorders. Please correct the statement (and citation) in the introductory section

---------------------------------------------

Reviewer 1

This Shank paper deals with the electrophysiological analysis of SC - CA1 synapses after lentivirus mediated knockdown of specific Shank family members. These experiments are important with respect to the analysis of the role Shank family members in the brain. Mutations of all (please correct the statement in the introductory statement accordingly, Leblond et al., 2014) family members can lead to neuropsychiatric diseases of the autism spectrum or to schizophrenia but the severity of the phenotype observed seems to be gene specific.

Major:

-The major question that arises is certainly the downregulation of Shank3 when Shank2 is specifically targeted by shRNA. The authors tackled that problem in part by overexpressing Shank3 in HEK cells and showing that these Shank3 levels are not altered by Shank2sh RNAs and that Shank3 levels can be rescued by Shank2 replacement. With respect to the missing experiment (Shank3 rescue by Shank3 lentiviral expression) authors argue that the size of Shank3 is too large.

It must be considered here that all Shanks are expressed in several different isoforms that are also brain region specific. Indeed Shank2 isoforms are more often starting with the SH3 domain or PDZ domain but there is also a Shank2 isoform (Shank2E) starting with the ankyrin repeats (therefore Fig. 1A is not correct in its present form). This Shank2 isoform is mainly expressed in the cerebellum but can also be found in minor amounts in the cortex.

Vice versa some Shank3 isoforms start with the SH3 domain or with the PDZ domain. These isoforms should be cloned into lentiviral vectors to perform the missing experiments (rescue with Shank3).

-The authors saw a especially a decrease of AMPA eEPSCs under certain KO conditions. It would strengthen the paper a lot, if these changes could also be seen on the protein level in Western Blot experiments on P2 fractions or by immunohistochemistry.

-The rescue experiments using Shank2 constructs with and without the SAM domain are nice but not really surprising having in mind that the SAM domain is needed for synaptic localization. Therefore, it would be a lot more informative to use several different Shank2 SAM domain containing isoforms expressing a different set of protein/protein interaction domains to analyse the most important region for rescue.

-What is puzzling is the analysis of control experiments in figure 1. It seems that some of the data obtained by the GFP control are significantly different from tdT (Shank1 expression). The authors should comment on this.

-The authors found a co-regulation of PSD95 with some of the Shank proteins. It would be important to know whether other excitatory PSD (Homer, Spar, Arc, SAPAPs....) components are also regulated or not. Therefore, the expression levels of other PSD proteins have to be tested in these experiments.

Minor:

-The list of cited literature appears to be not complete (multimerization of Shank, Shank and autism, isoforms of Shank proteins......)

-There are several citations (mentioned in the text) missing in the list of references....

Reviewer 2

This is a study aimed to investigate roles of different Shank isoforms at excitatory synapses. Using various single and combined shRNAs, the authors found that Shank1 and 2, but not Shank 3, play a major role in supporting excitatory synapse functions in hippocampal neurons. Overall the study is well constructed and experiments well executed. I have just a few points that require further clarification.

1. In Figure 1D, there are two versions of shShank2, the GFP-coexpressing one significantly reduced PSD95 expression, but not the tdTomato one. Are the shRNA sequences of these two shShank2 constructs the same? If the only difference between these two constructs is the coexpressing fluorophore, then what is the explanation of the differential effect on PSD95 expression?

2. Based on the evoked and mini EPSC data (Figure 3), the authors concluded that Shank 1 and Shank2 both are required for active synapses. What is the potential mechanism for this function of Shank1 and 2? Knocking down Shank 1 does not reduce PSD95 expression, but knockdown Shank 2 does. Therefore reduced PSD95 level cannot be the only explanation. Has the authors checked presynaptic release probability (i.e. paired-pulse ratio of eEPSC) as reduced mini frequency could be due to a reduction of Pr? Given that NMDARs have high affinity for glutamate, a small reduction in release probability may or may not show up as reduced NMDAR responses. It will be interesting if one (or both) Shank isoforms have a trans-syanptic effect on presynaptic function.

## Author Response

We appreciate the reviewers and ENeuro editors' constructive suggestion. Please find our response to specific questions below.

--------------------------------------------

Manuscript Instructions

- The species studied is not mentioned in the abstract. Please make sure to update both the abstract in the article file and on the submission form.

The species description is added in the abstract

- The sex of the species studied is not mentioned in the materials and methods section. Please mention “male”, “female”, “x males and x females”, “of either sex”.

The gender description is added in the materials and methods.

---------------------------------------------

Synthesis of Reviews:

Synthesis Statement for Author (Required):

The following issues are required to be addressed for a resubmission. I am also appending reviewer's comments at the end of this list, containing additional comments that authors might choose to address.

1. Authors should provide an explanation for the difference observed upon coexpressing shShank2 with GFP or td-Tomato.

We have run additional WBs, and reanalyzed the quantification. shShank2-GFP and shShank2-tdT do not show statistical significant differences.

2. Rescue experiments with small Shank3 isoforms (e.g., Shank3c, Shank3d) should be performed to aid in the interpretation of changes induced by sh-Shank2 expression. It would also be useful to determine if Shank2 can rescue the defects observed in Sh-Shank1.

We made constructs to express GFP-Shank3c in the background of shShank2 and shShank13, and found that Shank3c can rescue the synaptic deficit caused by knocking down Shank2 or Shank1.

3. Western blots or immunocytochemistry probing for potential changes in AMPAR levels should be included for in those conditions that alter AMPAR-mediated activity.

Example of WB using Anti-GriA1 and Anti-PanSAPAP antibodies are included in Supplemental Figure 2, with GFP, shShank2 and shShank2+Shank3 RP infected neuronal cultures P2 fraction. Data was not included in the MS, because this line of research requires extensive evidence before coming to meaningful conclusion about how Shank proteins influence synaptic composition. We think it is beyond the capacity of this MS.

4. Besides measurements of spontaneous release frequency, measurements of probability of release should be conducted.

Paired-pulse ratios were measured with uninfected, shShank1, shShank2, shShank3 and shShank1+shShank2. No significant difference was detected among different manipulations. (Figure 2E)

5. Authors should discuss the discrepancy between downregulation of Shank1 and Shank2 with regard to their effect on PSD95 and other non-PSD95 related potential mechanisms.

With the limited information on the discrepancy on PSD-95 levels with different manipulations. It will be difficult to draw concrete conclusion, or formulate potential mechanisms. We decided to take the observations on PSD-95 levels out of this MS, and investigate further in the future studies.

Minor issues:

1. Please carefully inspect the citation list, making sure that there is correspondence with citations within the text.

Reference updated.

2. The diagram in Figure 1A is not accurate as it neglects the depiction of alternative isoforms

Figure 1A updated, alternative isoforms were added. Annotation added in the figure to indicate this is not an exhaustive list of Shank isoforms

3. All Shank genes have been involved in psychiatric disorders. Please correct the statement (and citation) in the introductory section

Statement corrected and citation added. “All Shanks have been associated with neurological diseases such as schizophrenia and autism (Durand et al., 2007; Gauthier et al., 2010; Grabrucker et al., 2011b; Leblond et al., 2014), but the severity of the phenotype seems to be gene-specific.”

---------------------------------------------
